# Non-prescription sale of antibiotics and service quality in community pharmacies in Guangzhou, China: A simulated client method

**DOI:** 10.1371/journal.pone.0243555

**Published:** 2020-12-10

**Authors:** Lishan Kuang, Yizhuo Liu, Wei Wei, Xueqing Song, Xiaoqian Li, Qiqi Liu, Weimin Hong, Qian Liu, Jingwei Li, Zhongwei Chen, Yu Fang, Sujian Xia

**Affiliations:** 1 Department of Health Statistics, School of Medicine, Jinan University, Guangzhou, Guangdong, P.R. China; 2 School of Pharmacy, Jinan University, Guangzhou, Guangdong, P.R. China; 3 School of Pharmacy, Xi’an Jiaotong University, Xi’an, Shaanxi, P.R. China; The University of Sydney School of Pharmacy, AUSTRALIA

## Abstract

**Objective:**

To measure the situation of the non-prescription sale of antibiotics and the service quality of community pharmacies in Guangzhou, China.

**Methods:**

A simulated client method was conducted to estimate the non-prescription sale of antibiotics and service quality based on scenarios about adult acute upper respiratory tract infection in 2019. A total of 595 community pharmacies from 11 districts were investigated in Guangzhou, China. We used binary logistic regression to evaluate the factors associated with the non-prescription sale of antibiotics.

**Results:**

The proportion of non-prescription dispensing of antibiotics was 63.1% in Guangzhou, China, with a higher incidence of antibiotic dispensing without prescription in outer districts (69.3%). Cephalosporin (44.1%) and Amoxicillin (39.0%) were sold more often than other antibiotics. Chain pharmacies had better performance on the prescription sale of antibiotics and service quality. Traditional Chinese medicine was commonly recommended by pharmacy staff.

**Conclusion:**

Since the non-prescription sale of antibiotics is prevalent in Guangzhou, effective solutions should be determined. Strengthened public awareness and regulatory system innovation are needed.

## Introduction

Antibiotics are the most commonly prescribed medicines in the world [1]. Between 2000 and 2015, Global dispensing of antibiotics increased by 65% [2]. China was one of the leading consumers among low- and middle-income countries, where antibiotic consumption increased by 79% between 2000 and 2015 [2]. Due to the large sales of antibiotics, the issue of antibiotic resistance has drawn global attention because it can affect not only health but also the economy [3].

A prescription is a medical document issued by a licensed doctor to certify a patient’s medication [4]. The non-prescription sale of antibiotics reduces barriers to obtaining an antibiotic for patients, which may increase the sales of antibiotics. The global prevalence of the non-prescription sale of antibiotics urgently requires solutions, especially in developing countries [5]. As early as 2003, the National Medical Products Administration of China clearly stated that antibiotics that are not over-the-counter medicines must be sold with prescriptions issued by licensed physicians in all community pharmacies nationwide [6]. In 2016, the Chinese government proposed that all community pharmacies across the country dispense antibacterial drugs based on prescriptions by 2020 [7]. In Tanzania, the percentage (92.3%) of retailers dispensing antibiotics without a prescription was high and the description of drug side effects was limited [8]. Most respondents in Nigeria bought antibiotics to self-treat cold and cough [9]. A report from Zambia showed that most customers acquired antibiotics without a prescription in community pharmacies, which is similar to the findings from the Kingdom of Saudi Arabia [10, 11]. In India, among those patients who consumed antibiotics for the treatment of upper respiratory tract infections, only 7% of them have been prescribed a prescription from a licensed doctor [12]. The non-prescription sale of antibiotics grew rapidly in Hungary even though antibiotics can only be obtained by prescription [13]. A meta-analysis published in 2019 estimated the proportion of the non-prescription sale of antibiotics in community pharmacies worldwide [14]. In Asia, 65% of antibiotics were sold without a prescription in community pharmacies [14]. More than a half of pharmacy staff sold antibiotics without a prescription in surveyed areas of China [14].

Liu reported that it was unknown which antibiotics are dispensed most often without a prescription [5]. We found that Chang et al. [15] performed research on the state of non-prescription sales of antibiotics and trends in six provinces of China, which should be commended. However, the popularity of non-prescription sales of antibiotics in community pharmacies in southern China is still not known. As the province with the largest population in China, Guangdong Province has over 50,000 pharmacies [16, 17]. As the capital of Guangdong Province, there are 6,021 community pharmacies in Guangzhou city [18]. The status of the non-prescription sale of antibiotics in Guangzhou city should be given close consideration. In addition, respiratory diseases are one of the leading causes of death in Chinese urban residents [16]. Moreover, treatments of adult upper respiratory tract infections are commonly associated with the misuse of antibiotics by household self-medication [9, 12]. Chang et al. [15] also mention that the effect of pharmaceutical chains on service quality needs to be studied further. With the advancement of the Internet, pharmacy internet services are very efficient at some times, such as the period when people are required to maintain social distance to prevent the epidemic of COVID-19 [19].

In our study, investigators simulate patients with upper respiratory tract infections to inquire into the non-prescription sale of antibiotics and the service quality at community pharmacies in Guangzhou city. We explore the differences between urban and rural areas by comparing non-prescription sale of antibiotics of the main districts and the outer districts. The research results can provide a basis for the Guangzhou government to formulate regulations on promoting the prescription-based sale of antibiotics in community pharmacies. Moreover, we explore the current status and influencing factors of the service quality, including network services of community pharmacies in Guangzhou. Compared to sole-proprietor pharmacies, chain pharmacies may have more funds and a better employee training system. We expect that the chain pharmacies would perform better than sole-proprietor pharmacies in terms of service quality. Therefore, we compare the service quality of sole-proprietor pharmacies and chain pharmacies in this study.

## Method

### Study design

The simulated client method included investigators trained strictly in accordance with planned procedures to survey the service site as ordinary customers to simulate a real-world experience. In 2019, we conducted the simulated client method on 595 community pharmacies located in 11 districts in Guangzhou city, which is the capital of Guangdong Province. The sample size is approximately 10% of all pharmacies in Guangzhou city. The distribution of surveyed pharmacies is consistent with the distribution of the resident populations in each district. Please refer to S1 Appendix for the sample composition. Convenience sampling was done at each district. Since the government office are usually located in the center of community, we investigated pharmacies that are near the government office in each street of the district. During pre-survey, 50 pharmacies were randomly selected for investigator training and record sheet modifications. Eight pharmacy students from Jinan University conducted the investigation while simulating adult upper respiratory tract infections after three standard training sessions. Three levels of demand were used to obtain the antibiotic [15]: The pharmacy staff recommended antibiotics actively to the simulated client after the simulated client describes his/her symptoms (level 1); the antibiotic was sold when the simulated client asked for antibiotics (level 2); the antibiotic was sold when the simulated client asked for amoxicillin or cephalosporin (level 3). Please refer to S2 Appendix for the investigation process. The name and specific address of the pharmacy under investigation was not be recorded. Instead, a serial number was assigned to the record sheet.

### Study variables

The record sheet that was modified based on published studies consisted of four parts [15] (S3 Appendix). First, characteristics of pharmacies were recorded including pharmacy scale, pharmacy type, pharmacist license, prescription drug sign, prescription drug counter, is the pharmacist on duty, whether to provide online services and whether it is a medical insurance designated pharmacy and so on. Second, characteristics of the reception staff observed during investigation were also recorded, including gender, age and whether the reception staff was a licensed pharmacist. Third, the non-prescription sale of antibiotics by investigated pharmacies and the category of antibiotics sold without acquiring prescription was recorded. Finally, the service quality of community pharmacies was recorded. In this part, we recorded whether or not reception staff asked about a history of drug allergy during the non-prescription sale of antibiotics, whether or not patients were asked about other drugs being taken, and whether or not patients were advised to see a doctor, given advice regarding medication or prevention, or given recommendations for medicines other than antibiotics. Furthermore, service time, whether or not service was provided online, and any specific advice given by staff, including the specific name of non-antibiotic medicines recommended by staff, were recorded.

### Statistical analysis

It is reported that the proportion of non-prescription sales of antibiotics is higher in rural areas [20]. Outer districts have a larger rural population than main districts. For that reason, we compared the results of main districts and outer districts. Univariate analyses were conducted to compare characteristics of pharmacies, characteristics of reception staff, the non-prescription sale of antibiotic, the category of antibiotics sold and the service quality of community pharmacies between the main district and the outer district. We performed multivariate, binary logistic regressions to evaluate the factors associated with the non-prescription sale of antibiotics in the main and outer districts adjusted for the age and gender of reception staff [15]. Differences in service quality between sole-proprietor pharmacies and chain pharmacies were also analyzed. Chi-squared tests were used to compare categorical variables between two groups. Additionally, we compared the percentage of medication or prevention advice provided from staff and medicines other than antibiotics recommended by staff between the main districts and outer districts. A p-value of < 0.05 was considered significant. Pairwise deletion method, which is considered effective when only a small part of data are missing, was used to handle missing data [21].

This study was approved by the Institutional Review Board of the First Affiliated Hospital of Jinan University. We used the simulated client method, as the study cannot be performed if the investigators’ identity is disclosed to the pharmacy staff. Since it is an observational and minimal-risk study, consent from pharmacies was waived under the policy of the IRB.

## Results

### Characteristics of pharmacies and reception staff

Table 1 shows the characteristics of pharmacies and reception staff. The total pharmacies surveyed numbered 595, consisting of 406 pharmacies from the main districts and 189 pharmacies from the outer districts. Among all pharmacies, over 70% of pharmacies were considered medium in scale. No significant difference was found between the main districts and the outer districts. Compared to the outer districts, the main districts had more chain pharmacies (*p* = 0.002). Almost 90% of pharmacies both in the main districts and the outer districts had signed a medical insurance agreement with the Medical Insurance Agency. In other words, the cost of the applicant buying medicines in these pharmacies was partly covered by medical insurance. The great majority of pharmacies had a pharmacist license, a sign of prescription drug and a prescription drug counter. Only approximately one-third of pharmacies had a pharmacist on duty. In terms of the characteristics of reception staff, few reception staff were registered pharmacist. The gender of most of reception staff was female. More than half were between 30 and 50 years old. Relative to the outer districts, the reception staff were younger in the main districts (*p* <0.001).

**Table 1:** The characteristics of community pharmacies and reception staff.

Characteristics	Total	Main districts	Outer districts	*P*
	n(%)	n(%)	n(%)	
**Number of pharmacies**	595(100.0)	406(62.2)	189(31.8)	
characteristics of pharmacies				
**Pharmacy scale** ^**1**^				0.284
Large	82(13.8)	53(13.1)	29(15.3)	
Medium	422(71.0)	287(70.9)	135(71.4)	
Small	90(15.2)	65(16.1)	25(13.2)	
**Pharmacy type** ^**1**^				
Sole-proprietor	100(16.8)	55(13.6)	45(23.9)	0.002
Chain	494(83.2)	351(86.5)	143(76.1)	
**Medical insurance** ^**1**^				
Yes	525(88.4)	357(87.9)	168(89.4)	0.613
No	69(11.6)	49(12.1)	20(10.6)	
**Pharmacist license** ^**2**^				0.085
Yes	523(88.2)	350(86.6)	173(91.5)	
No	70(11.8)	54(13.4)	16(8.5)	
**Sign of Prescription drug** ^**1**^				0.709
Yes	576(97.0)	392(96.8)	184(97.4)	
No	18(3.0)	13(3.2)	5(2.7)	
**Prescription drug counter**				0.377
Yes	577(97.0)	392(96.6)	185(97.9)	
No	18(3.0)	14(3.5)	4(2.2)	
**Pharmacist on duty** ^**3**^				0.819
Yes	204(34.6)	138(34.3)	66(37.3)	
No	385(65.4)	264(65.7)	121(62.7)	
characteristics of reception staff				
**Pharmacist** ^**4**^				0.499
Yes	126(21.3)	83(20.5)	43(23.0)	
No	465(48.7)	321(79.5)	144(77.0)	
**Gender**				0.496
Male	98(16.5)	64(15.8)	34(18.0)	
Female	497(83.5)	342(84.2)	155(82.0)	
**Age (years)**				<0.001
<30	235(39.5)	180(44.3)	55(29.1)	
30–50	334(56.1)	212(52.2)	122(64.6)	
>50	26(4.4)	14(3.5)	12(6.4)	

Note. Variables ^1^ had 0.2% missing values; Variables ^2^ had 0.3% missing values; Variables ^3^ had 1.0% missing values; Variables ^4^ had 0.7% missing values. Pairwise deletion was used to handle missing data.

### Non-prescription sale of antibiotics

The non-prescription sale of antibiotics in Guangzhou is summarized in Table 2. A total of 375/595 (63.1%) interactions resulted in the non-prescription sale of antibiotics. Significant differences were found in the non-prescription sale of antibiotics between the main districts and the outer districts. Pharmacies in outer districts were more likely to distribute antibiotics without requiring a prescription. A total of 72 (12.1%) pharmacies sold antibiotics under demand level 1. A total of 127 (21.4%) pharmacies sold antibiotics under demand level 2. Cephalosporin and Amoxicillin were most often sold when reception staff sold antibiotics actively (level 1) and investigators asked for an antibiotic during interactions of the non-prescription sale of antibiotics (level 2). The category of antibiotics sold without prescription was not significantly different between the main districts and the outer districts.

**Table 2:** Non-prescription sale of antibiotics in Guangzhou.

Characteristics	Total	Main districts	Outer districts	*P*
	n(%)	n(%)	n(%)	
**Non-prescription sale of antibiotic** ^**1**^				0.033
Yes	375(63.1)	244(60.3)	131(69.3)	
No	219(36.9)	161(39.8)	58(30.7)	
**The category of antibiotics sold** ^**2**^				0.372
Cephalosporin	86(44.1)	53(43.8)	33(44.5)	
Amoxicillin	76(39.0)	46(38.0)	30(40.5)	
Roxithromycin	9(4.6)	6(5.0)	3(4.1)	
Azithromycin	13(6.7)	11(9.1)	2(2.7)	
Other	11(5.6)	5(4.1)	6(8.1)	

Note. Variables ^1^ had 0.2% missing values; Variables ^2^ had 3.0% missing values. Pairwise deletion was used to handle missing data. The category of antibiotics sold was recorded when reception staff sold antibiotics actively and investigators asked for an antibiotic in interactions of the non-prescription sale of antibiotics.

### Univariate analysis and multiple logistic regression analysis

Table 3 shows that a univariate logistical analysis was used to ascertain the relationship between the non-prescription sale of antibiotics and characteristics of pharmacies and reception staff. Among those characteristics, pharmacy scale and pharmacy type were significantly associated with the non-prescription sale of antibiotics for pharmacies located in the main districts. Medium (*p* = 0.0369) and small (*p* = 0.0365) pharmacies were more inclined to dispense antibiotics without a prescription than large pharmacies. Additionally, sole-proprietor pharmacies had a greater proportion of non-prescription sales of antibiotics than chain pharmacies. We calculated the adjusted OR by adjusting for gender and the age of reception staff (S4 Appendix). The multiple regression model showed that pharmacy scale was related to the non-prescription sale of antibiotics for pharmacies located in the main districts (*p*<0.05). No association between the non-prescription sale of antibiotics and the characteristics of pharmacies and reception staff in outer districts was observed.

**Table 3:** The relationship between the non-prescription sale of antibiotics and the characteristics of pharmacies and reception staff in Guangzhou.

Survey project	Main districts		Outer districts	
	OR (95% CI)	*p*	OR (95% CI)	*p*
characteristics of pharmacies				
**Pharmacy scale**				
Large	1		1	
Medium	1.89(1.04,3.42)	0.0369	0.87(0.35,2.13)	0.755
Small	2.23(1.05,4.72)	0.0365	0.85(0.26,2.75)	0.786
**Pharmacy type**				
Sole-proprietor	1		1	
Chain	0.52(0.27,0.99)	0.0471	1.68(0.84,3.39)	0.145
**Medical insurance**				
Yes	1		1	
No	1.52(0.80,2.90)	0.207	0.82(0.31,2.19)	0.697
**Pharmacist license**				
Yes	1		1	
No	0.78(0.43,1.43)	0.423	0.66(0.22,1.94)	0.445
**Sign of Prescription drug**				
Yes	1		1	
No	1.78(0.46,6.80)	0.401	0.67(0.11,4.13)	0.668
**Prescription drug counter**				
Yes	1		1	
No	0.65(0.21,2.05)	0.46	0.44(0.06,3.24)	0.423
**Pharmacist on duty**				
Yes	1		1	
No	1.46(0.96,2.22)	0.081	1.45(0.76,2.74)	0.259
characteristics of staff				
**Gender**				
Male	1		1	
female	0.81(0.46,1.43)	0.466	0.51(0.21,1.26)	0.145
**Age (years)**				
<30	1		1	
30–50	1.16(0.77,1.75)	0.490	0.74(0.37,1.50)	0.404
>50	0.70(0.24,2.08)	0.520	1.13(0.27,4.72)	0.872
**Pharmacist**				
Yes	1		1	
No	1.06(0.64,1.74)	0.821	1.85(0.91,3.76)	0.0871

### Service quality

**Fig 1:**
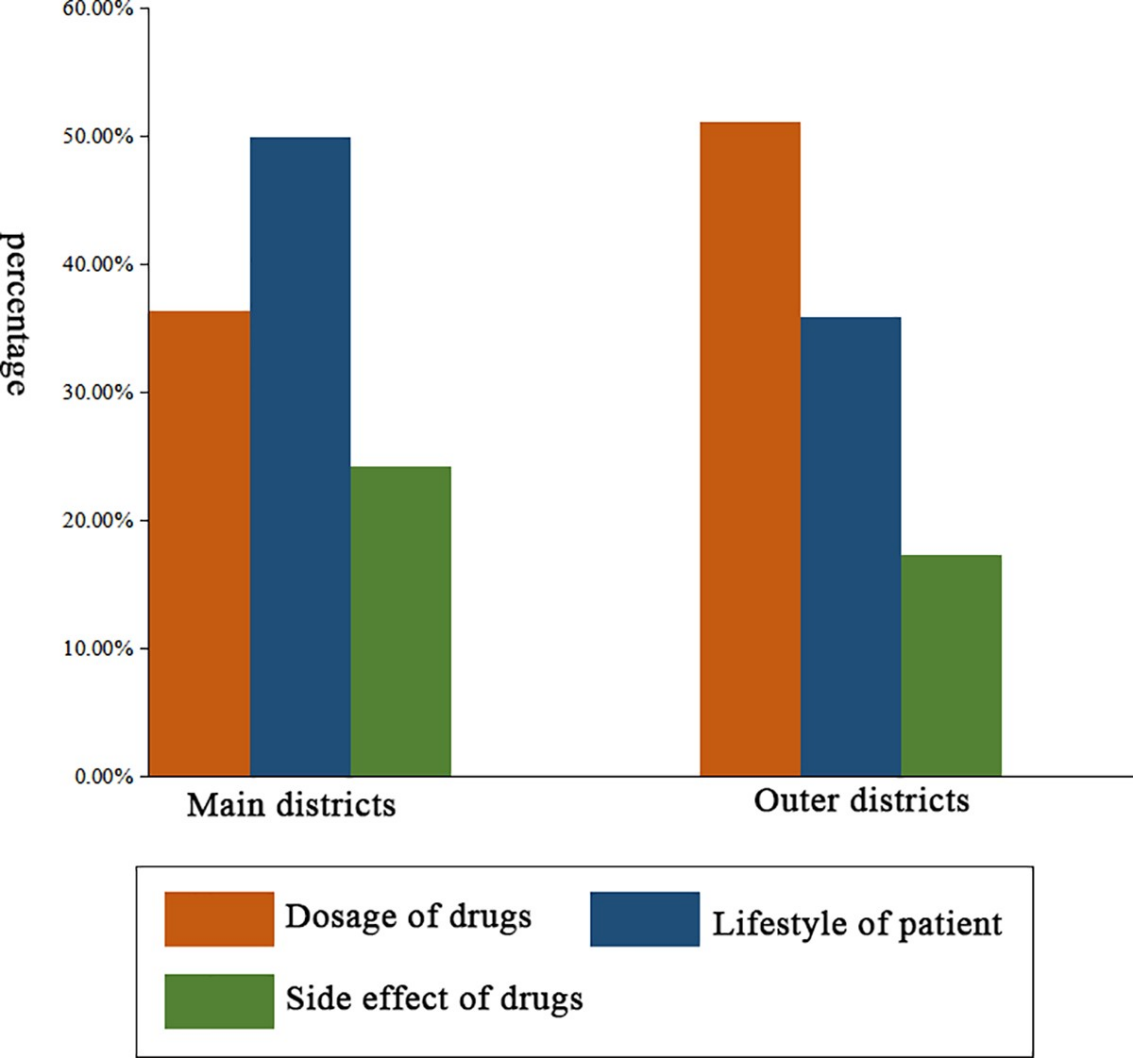
Medication or prevention advice given by reception staff in community pharmacies in Guangzhou.

**Fig 2:**
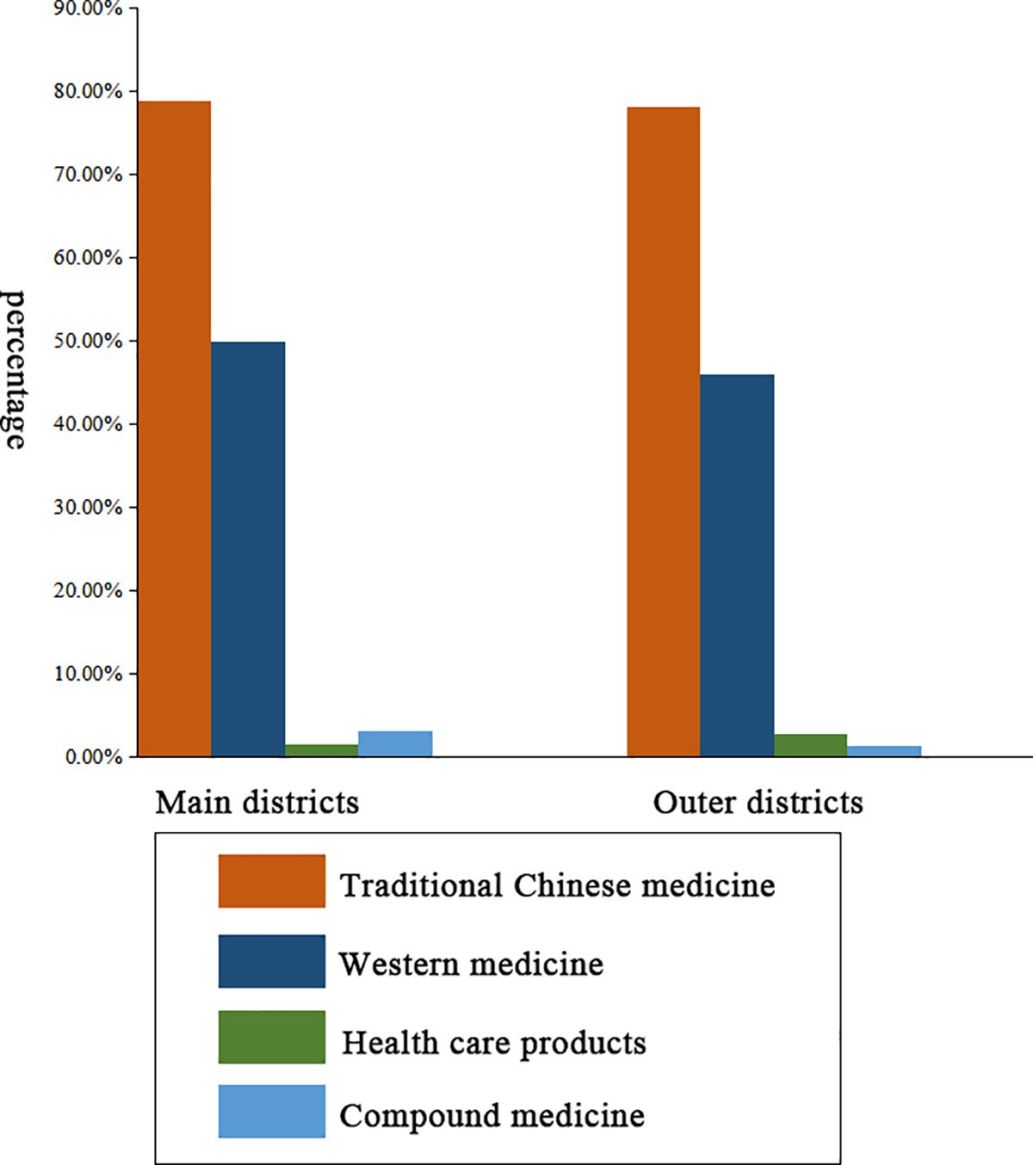
The types of non-antibiotic medicines recommended by reception staff in community pharmacies in Guangzhou.

First, we compared the service quality of pharmacies between the main districts and the outer districts, as shown in Table 4. Suggestions about medication or prevention were given in 47.3% and 48.7% of interactions when the non-prescription sale of antibiotics happened in the main districts and the outer districts, respectively (Fig 1). Reception staff of pharmacies in the main districts tended to offer advice about lifestyle. Comparatively, suggestions on the dosage of drugs were more recommended in the outer districts. A majority of reception staff recommended drugs other than antibiotics (Fig 2). Among those interactions, the most popular category of drugs was traditional Chinese medicine. No significant differences between the main and outer districts were observed in whether or not the staff asked about a history of drug allergies, whether or not suggestions about medications or preventions were given, whether or not a list of current medications was solicited, whether or not it was suggested to go to the doctor and whether or not online services were provided (*p*>0.05).

**Table 4:** Differences in the service quality of community pharmacies between the main districts and the outer districts in Guangzhou.

Characteristics	Total	Main districts	Outer districts	*P*
	n(%)	n(%)	n(%)	
**Online service**				0.549
Yes	40(6.7)	29(7.1)	11(5.8)	
No	555(93.3)	377(92.9)	178(94.2)	
**Service time**				0.437
<5 min	471(79.2)	327(80.5)	144(76.2)	
5–10 min	122(20.5)	78(19.2)	44(23.3)	
>10 min	2(0.3)	1(0.3)	1(0.5)	
**Given medication or prevention advice** ^**1**^				0.750
Yes	283(47.7)	191(47.3)	92(48.7)	
No	310(52.3)	213(52.8)	97(51.3)	
**Recommended medicines other than antibiotics**				0.329
Yes	584(98.2)	397(97.8)	187(98.9)	
No	11(1.9)	9(2.2)	2(1.1)	
**Asked about a history of drug allergy in the non-prescription sale of antibiotics** ^**2**^				0.395
Yes	181(48.4)	122(50.0)	59(45.4)	
No	193(51.6)	122(50.0)	71(54.6)	
**Asked whether the patient had taken other drugs or not** ^**3**^				0.117
Yes	39(6.6)	31(7.7)	8(4.3)	
No	552(93.4)	372(92.3)	180(95.7)	
**Advised patient to see a doctor** ^**1**^				0.311
Yes	19(3.2)	15(3.7)	4(2.1)	
No	574(96.8)	390(96.3)	184(98.9)	

Note. Variables ^1^ had 0.3% missing values; Variables ^2^ had 0.3% missing values; Variables ^3^ had 0.7% missing values. Pairwise deletion was used to handle missing data.

Second, the results of the service quality of sole-proprietor pharmacies and chain pharmacies are shown in Table 5. We found statistically significant differences in asking for a history of drug allergies or not and in providing online service or not between sole-proprietor pharmacies and chain pharmacies (*p*<0.05). In the interaction of the non-prescription sale of antibiotics, more reception staff in chain pharmacies asked whether the patient had a drug allergy history. Moreover, the proportion of chain pharmacies that had an online service platform is larger than that for sole-proprietor pharmacies.

**Table 5:** Differences of in service quality between sole-proprietor pharmacies and chain pharmacies in Guangzhou.

Characteristics	Sole-proprietor pharmacies	Chain pharmacies	*P*
	n(%)	n(%)	
**Asked about a history of drug allergy in the non-prescription sale of antibiotic**			0.033
Yes	24(36.4)	156(50.8)	
No	42(63.6)	151(49.2)	
**Asked whether the patient had taken other drugs or not**			0.270
Yes	4(4.1)	35(7.1)	
No	94(95.9)	457(92.9)	
**Advised patient to see a doctor**			0.912
Yes	3(3.0)	16(3.3)	
No	390(96.4)	477(96.8)	
**Online service**			0.012
Yes	1(1.0)	39(7.9)	
No	99(99.0)	455(92.1)	
**Service time**			0.399
<5 min	83(83.0)	387(78.3)	
5–10 min	16(16.0)	106(21.5)	
>10 min	1(1.0)	1(0.2)	
**Given medication or prevention advice**			0.094
Yes	40(40.0)	242(49.2)	
No	60(60.0)	250(50.8)	
**Recommended medicines other than antibiotics**			0.081
Yes	96(96.0)	487(98.6)	
No	4(4.0)	7(1.4)	

## Discussion

In our study, more than half of community pharmacies in Guangzhou dispensed antibiotics without a prescription. The proportion of the non-prescription sale of antibiotics in community pharmacies in main districts is significantly less than that in community pharmacies in outer districts. For community pharmacies in main districts, scale and type are influencing factors in the non-prescription sale of antibiotics. On the other hand, we also investigated the service quality of community pharmacies. We found that only a few pharmacies can provide internet services. The service hours of community pharmacies are generally short. Almost all reception staff recommended medicines other than antibiotics to patients. Approximately half of the pharmacy reception staff did not ask customers about their drug allergy history. Compared to the sole-proprietor pharmacies, chain pharmacies have better performance in service quality.

The proportion of the non-prescription sale of antibiotics in Guangzhou, China (63.1%) is equivalent to the pooled estimate globally (62%, 95% CI 53–72) [14]. Additionally, the percentage of Guangzhou is lower than the pooled estimate of South America (78%, 95CI% 72–84) and Africa (74%, 95% CI 62–85) [14]. A survey done in 2019 [15] found that the proportion of non-prescription sales of antibiotics in eastern, central, and western China is 70.1%, which is higher than the proportion in Guangzhou found here. The results present here indicate that pharmacies in the outer districts were more likely to sell antibiotics without a prescription than those in the main districts. We think that the difference between the main districts and outer districts may be due to the following reasons. First, the outer districts have a less developed economy than the main districts [22]. Individuals with poor financial ability are more likely avoid consulting doctors due to considerations of expense and thus choose self-treatment [23]. Second, the number of villages in the outer districts is relatively large [24]. Individuals in rural areas have less knowledge about antibiotics [25]. Moreover, the non-prescription sale of antibiotics is more likely happen in rural than urban areas [20]. Therefore, pharmacies in outer districts may more routinely sell antibiotics without acquiring a prescription.

In keeping with work done in India and other provinces of China, our study shows that factors that affect the non-prescription sale of antibiotics in the main districts are the scale and type of pharmacy [15, 26]. Chain pharmacies located in the main districts are more inclined to sell antibiotics with a prescription from a doctor. Medium and small pharmacies are associated with more non-prescription sales of antibiotics than large pharmacies. Smaller pharmacies and sole-proprietor pharmacies may have relatively little funding. For that reason, staff training may not be carried out effectively in smaller pharmacies. At the same time, factors affecting the non-prescription sale of antibiotics in outer districts was not found. However, the illegal rate of pharmacies in outer districts is higher than that in the main districts. Therefore, the factors that affect the proportion of non-prescription sales of antibiotics by pharmacies in outer districts are not the characteristics of the pharmacies we studied but may be due to other reasons. The specific reasons that affect the proportion of non-prescription sales of antibiotics of the pharmacies in outer districts needs further research.

In our study, when reception staff sold antibiotics actively or investigators asked for an antibiotic, Cephalosporin and Amoxicillin were most often sold. Amoxicillin was also found to be the most commonly non-prescribed antibiotic sold in Zambia [10]. Cephalosporin and Amoxicillin are commonly used drugs to treat infectious diseases in many countries, such as the United States, the Netherlands and France [27–29]. Cephalosporin should be used as a reserve drug rather than the first choice for the treatment of infectious diseases. For that reason, the high proportion of non-prescription sales of Cephalosporin should be taken seriously. Additionally, allergic reactions to Amoxicillin and Cephalosporin, which are both beta-lactams, are commonly reported [30]. However, less than half of reception staff asked if the patient had a history of drug allergies when they sold antibiotics without a prescription in our study. Additionally, similar to a study in Tanzania, our work found that side effects of medicines are rarely mentioned [8]. Beta-lactam allergies may cause bronchospasm and even anaphylactic shock in some severe cases [30]. Hence, staff training about medication specification should be strengthened. We believe that the risk of patient antibiotic abuse could be reduced when the allergy history of the patient is known to reception staff.

As we observed, traditional Chinese medicine was popular with reception staff in the treatment of adult colds. In recent years, traditional Chinese medicine has shown some effectiveness in the relief of clinical symptoms in the treatment of adult acute respiratory tract infections and some non-infectious diseases [31–33]. However, well-designed experiments are needed to further prove the efficacy and safety of traditional Chinese medicine [31].

In addition to non-prescription sales of antibiotics, we analyzed the quality of service in pharmacies. Service times at community pharmacies in Guangzhou were generally less than five minutes. In this short time, reception staff may only ask the main symptoms of a patient. Some situations that needed to be noticed may be ignored, such as a history of drug allergies of patients. Therefore, a lack of information from patients may lead to reception staff making an incorrect decision on medication dispensation. Chain pharmacies have sufficient funds, so the content of employee training may be more abundant. Compared with Sole-proprietor pharmacies, the proportion of employees of chain pharmacies that made recommendations on disease prevention may be greater. Therefore, in the main districts with more chain pharmacies, more staffs advise patients to change their lifestyles to prevent disease or its deterioration. Chain pharmacies have better performance in service quality. The reception staff of chain pharmacies were more likely to ask about the allergy history of the patient, which means that chain pharmacies can provide safer medication services to patients than sole-proprietor pharmacies. Additionally, a greater proportion of chain pharmacies provide online service. Online pharmacies have enormous potential in the future [34]. The global market of online pharmacies was approximately USD 42.3 billion in 2018; this is estimated to expand to USD 107.5 billion in 2025 [35]. According to a study of seven countries, using online health services has resulted in more relaxation than anxiety [36]. As research published in 2016 reported, a quarter of respondents searched for information on a medication online [37]. Online pharmacies can facilitate individuals with reduced mobility and individuals living in remote areas. Moreover, medicine sales data can be quickly and accurately collected from a unified online pharmacy platform. Real-time medicine sales data enable regulators to monitor antibiotic dispensing efficiently. It is feasible to establish a unified pharmacy drug sales platform in China [38]. However, a regulatory system on online pharmacies also needs to be established because it is also possible to sell antibiotics without a prescription online [39, 40].

Some published studies show that a pharmacist’s participation in intervention design, education for dispensing staff, public education campaigns and supervision from government staff are effective in reducing the probability of non-prescription sales of antibiotics [41]. Moreover, interventions should continue to be implemented for a sufficiently long duration [42]. In other words, dispensers and the public should be regularly educated. Government personnel should regularly carry out supervision of the non-prescription sale of antibiotics.

Raising caution with the public and pharmacists regarding the prescription of antibiotics is necessary. In developing countries, the proportion of self-treatment with antibacterial drugs was 38.8% [43]. Research performed in Chinese cities showed that 41.0% of caregivers were not aware that a doctor’s prescription is necessary to buy antibiotics from community pharmacies [44]. Thus, to reduce public demand for antibiotics without a prescription, public education on regulations of prescription drugs should be strengthened. Effective education can be achieved not only through traditional paper media but also through internet media. More diversified ways of disseminating information on the Internet should be considered, such as video, music, and images. More importantly, training on antibiotic medications for pharmacy workers is key to reducing antibiotic abuse. Pharmacists have insufficient knowledge of the efficacy of antibiotics [45]. To update the knowledge of pharmacists in a timely manner, routine testing should be held in addition to the licensed pharmacist qualification examination.

Laws and regulations on antibiotic medication need to be improved and implemented effectively. Poor regulations and a lack of qualified pharmacists were contributing factors for the non-prescription sales of antibiotics in developing countries [46]. Additionally, as reported previously, licensed pharmacists on duty are associated with the prescription sale of antibiotics [15]. According to the regulations, community pharmacies must be equipped with licensed pharmacists [47]. However, in China, the scale of licensed pharmacists is still insufficient [48]. Moreover, no pharmacist is actually on duty in a large number of pharmacies [49]. Fines have proven effective in reducing non-prescription sales of antibiotics [50]. Training more licensed pharmacists and increasing penalties for illegal community pharmacies could be effective methods.

Our study has some limitations. First, although uniform training for investigators was conducted, differences between simulated customers may still exist. The symptoms simulated by different investigators may vary. Second, we sampled pharmacies according to the distribution of the number of permanent residents and implemented convenience sampling in each district in Guangzhou city. Pharmacies investigated by simulated customers may be more inclined to comply with regulations because those pharmacies are geographically close to the local government position. In other words, the proportion of the non-prescription sale of antibiotics in our study may be underestimated. Third, the sample size of this study is approximately 10% of all pharmacies in Guangzhou city. The data for the total number of pharmacies in Guangzhou comes from a study published in 2011. As the population increases, the total number of pharmacies in Guangzhou may increase during the intervening years. Therefore, the actual sample size of this study may be smaller than mentioned above. The representativeness of the sample may be overestimated. Fourth, as an observational study, this study cannot explore the effect of interventions on the non-prescription sale of antibiotics in Guangzhou city. Fifth, according to the above research results, the factors affecting the non-prescription sale of antibiotics in outer districts have not been found. Further research can explore the impact of other factors (such as different diseases, knowledge, attitude and perception of pharmacy staff) on the non-prescription sale of antibiotics in outer districts [51, 52]. Last, three levels of demand were used to obtain the antibiotics in our study. The customer made a request to buy antibiotics in level 2 and level 3, which may make pharmacies more inclined to sell antibiotics without prescription.

## Conclusion

Our findings fill a data gap regarding the non-prescription sale of antibiotics in Guangzhou, China. Non-prescription sales of antibiotics in Guangzhou were less severe than in other regions studied in China but were still prevalent. Cephalosporin and Amoxicillin were sold more often than other antibiotics. The frequent use of Cephalosporin and Amoxicillin should be paid close attention. Drug side effects were rarely described clearly. A higher incidence of antibiotic dispensation without a prescription was observed in outer districts. Chain pharmacies had better performances on the prescription sale of antibiotics and service quality, including inquiring about the allergy history of the patient and providing online service. Traditional Chinese medicine was commonly recommended by pharmacy staff. Public awareness and pharmacist training should be strengthened.

## Supporting information

S1 Appendix Sample composition. (DOCX)

S2 Appendix Investigation process. (DOCX)

S3 Appendix Recording sheet. (DOCX)

S4 Appendix The relationship between the non-prescription sale of antibiotics and the characteristics of pharmacies and reception staff in Guangzhou. (DOCX)
