# Supplementary material for: Non-prescription sale of antibiotics and service quality in community pharmacies in Guangzhou, China: A simulated client method
Source: PLoS One. 2020 Dec 10;15(12):e0243555. doi: 10.1371/journal.pone.0243555 (PMC7728288; doi:10.1371/journal.pone.0243555)
Supplement: S1 Appendix — (DOCX) [file pone.0243555.s001.docx]

**S1 Appendix. Sample composition**

| Districts | Permanent population^1^ | |  | Theoretical sample size | |  | Actual sample size | |
| --- | --- | --- | --- | --- | --- | --- | --- | --- |
|  | Population(* 10 thousand) | Proportion |  | Sample size | Proportion |  | Sample size | Proportion |
| Baiyun | 257.2 | 17.7% |  | 106 | 17.7% |  | 97 | 16.3% |
| Panyu | 171.9 | 11.9% |  | 71 | 11.8% |  | 74 | 12.4% |
| Haizhu | 166.3 | 11.5% |  | 69 | 11.5% |  | 57 | 9.6% |
| Huangpu | 166.3 | 11.5% |  | 45 | 7.5% |  | 40 | 6.7% |
| Liwan | 95.0 | 6.6% |  | 39 | 6.5% |  | 50 | 8.4% |
| Tianhe | 169.8 | 11.7% |  | 70 | 11.7% |  | 79 | 13.3% |
| Yuexiu | 116.4 | 8.0% |  | 48 | 8.0% |  | 49 | 8.2% |
| Zengcheng | 119.8 | 8.3% |  | 50 | 8.3% |  | 49 | 8.2% |
| Huadu | 119.8 | 8.3% |  | 45 | 7.5% |  | 39 | 6.6% |
| Conghua | 64.2 | 4.4% |  | 27 | 4.5% |  | 29 | 4.9% |
| Nansha | 72.5 | 5.0% |  | 20 | 5.0% |  | 32 | 5.4% |
| Total | 1447.8 | 100.0% |  | 600 | 100.0% |  | 595 | 100.0% |

Note. ^1^Data comes from Population size and Distribution of Guangzhou in 2018.
