# Supplementary material for: Non-prescription sale of antibiotics and service quality in community pharmacies in Guangzhou, China: A simulated client method
Source: PLoS One. 2020 Dec 10;15(12):e0243555. doi: 10.1371/journal.pone.0243555 (PMC7728288; doi:10.1371/journal.pone.0243555)
Supplement: S2 Appendix — (DOCX) [file pone.0243555.s002.docx]

**S2 Appendix. Investigation process**

Observation of the pharmacy environment.

The simulated client introduces the patient's symptoms to the pharmacy staff.

The simulated client ask for a specific antibiotic.

The simulated client ask for antibiotics.

Pharmacy staff recommends medicine.

The simulated client leaves the pharmacy.

The simulated client fills in the record sheet.

Antibiotics are recommended

Antibiotics are recommended

No antibiotics provided

The staff refuse to provide antibiotic.

The staff provide antibiotics or still refuse to.
