# Supplementary material for: Non-prescription sale of antibiotics and service quality in community pharmacies in Guangzhou, China: A simulated client method
Source: PLoS One. 2020 Dec 10;15(12):e0243555. doi: 10.1371/journal.pone.0243555 (PMC7728288; doi:10.1371/journal.pone.0243555)
Supplement: S3 Appendix — (DOCX) [file pone.0243555.s003.docx]

**S3 Appendix.** **Recording sheet**

No.

**Investigator's name: ___________**

**Symptom: Adult upper respiratory tract infections.**

| **No.** | **Items** | **Results** | |
| --- | --- | --- | --- |
| **1** | Pharmacy scale | □Large（>100 m^2^） □Medium（50-100 m^2^）□Small（<50m^2^） | |
| **2** | Pharmacy type | □Sole-proprietor □Chain | |
| **3** | Does the pharmacy have signed a medical insurance agreement with the Medical Insurance Agency? ^*^ | □Yes □No | |
| **4** | Does the pharmacy have a clear label for selling prescription drugs? | □Yes □No | |
| **5** | Does the pharmacy have a clear display of pharmacist license? ^*^ | □Yes □No | |
| **6** | Does the pharmacy have an independent prescription drug counter? ^*^ | □Yes □No | |
| **7** | Is a licensed pharmacist on duty? | □Yes □No | |
| **8** | Whether the receptionist is a licensed pharmacist? | □Yes □No | |
| **9** | Receptionist's gender | □ Male □ Female | |
| **10** | Receptionist's age | □<30 years □ 30-50 years □ >50 years | |
| **11** | Whether the receptionist asked the patient about the detailed condition? | □Yes □No | |
| **12** | Does the pharmacy require customer to provide prescriptions to sell antibiotics？ | □Yes □No | |
| **13** | Does the pharmacy provide internet services? ^*^ | □Yes □No | |
| **14** | When antibiotic can be sold by pharmacies without a prescription, at what stage do pharmacy sell antibiotic? | □Stage1: The receptionist actively recommends antibiotics to customer.  □Stage2: The customer asks the receptionist to buy antibiotics.  □Stage3: The customer made a request to the customer to buy a specific antibiotic. | |
| **15** | Generic names of antibiotics sold: |  | |
| **16** | Does the receptionist ask the customer some medication questions? | 1. Is the customer taking other medicines? □Yes □No 2. Does the customer have a history of drug allergies? □Yes □No | |
| **17** | Does the receptionist advise the customer to visit the doctor? | □Yes □No | |
| **18** | Does the receptionist provide advice to the customer on medication or disease prevention? | □Yes  □No | □Dosage of drugs^*^  □Lifestyle of patient^*^  □Side effect of drugs^*^ |
| **19** | Reception time: | □ 0~5 min □ 5~10 min □ >10 min | |
| **20** | Does the receptionist recommend medicines other than antibiotics to customers? | □Yes  □No | □Traditional Chinese medicine^*^  □Western medicine^*^  □Health care products^*^  □Compound medicine^*^ |

Note. Item ^*^ is an added item.
