# Supplementary material for: Non-prescription sale of antibiotics and service quality in community pharmacies in Guangzhou, China: A simulated client method
Source: PLoS One. 2020 Dec 10;15(12):e0243555. doi: 10.1371/journal.pone.0243555 (PMC7728288; doi:10.1371/journal.pone.0243555)
Supplement: S4 Appendix — (DOCX) [file pone.0243555.s004.docx]

**S4 Appendix. The Relationship between the Non-Prescription Sale of Antibiotics and the Characteristics of Pharmacies and Reception Staff in Guangzhou.**

| Survey project | Main districts | | Outer districts | |
| --- | --- | --- | --- | --- |
|  | Adjusted OR (95% CI) | Adjusted *p* | Adjusted OR (95% CI) | Adjusted *p* |
| characteristics of pharmacies |  |  |  |  |
| **Pharmacy scale** |  |  |  |  |
| Large | 1 |  | 1 |  |
| Medium | 1.87(1.03,3.41) | 0.0404 | 0.82(0.33,2.06) | 0.675 |
| Small | 2.27(1.06,4.83) | 0.0342 | 0.82(0.24,2.78) | 0.744 |
| **Pharmacy type** |  |  |  |  |
| Sole-proprietor | 1 |  | 1 |  |
| Chain | 0.52(0.27,1.00) | 0.0512 | 1.68(0.81,3.48) | 0.166 |
| **Medical insurance** |  |  |  |  |
| Yes | 1 |  | 1 |  |
| No | 1.53(0.80,2.95) | 0.201 | 0.74(0.26,2.09) | 0.564 |
| **Pharmacist license** |  |  |  |  |
| Yes | 1 |  | 1 |  |
| No | 0.78(0.42,1.42) | 0.412 | 0.55(0.17,1.79) | 0.317 |
| **Sign of Prescription drug** |  |  |  |  |
| Yes | 1 |  | 1 |  |
| No | 1.91(0.49,7.39) | 0.348 | 0.76(0.11,5.06) | 0.772 |
| **Prescription drug counter** |  |  |  |  |
| Yes | 1 |  | 1 |  |
| No | 0.68(0.21,2.17) | 0.516 | 0.52(0.069,3.95) | 0.528 |
| **Pharmacist on duty** |  |  |  |  |
| Yes | 1 |  | 1 |  |
| No | 1.50(0.98,2.30) | 0.0604 | 1.51(0.78,2.92) | 0.224 |
| characteristics of staff |  |  |  |  |
| **Gender** |  |  |  |  |
| Male | - | - | - | - |
| female | - | - | - | - |
| **Age (years)** |  |  |  |  |
| ＜30 | - | - | - | - |
| 30-50 | - | - | - | - |
| >50 | - | - | - | - |
| **Pharmacist** |  |  |  |  |
| Yes | 1 |  | 1 |  |
| No | 1.14(0.67,1.91) | 0.635 | 2.07(0.97,4.42) | 0.0618 |
